# Natural Convection Water/Glycerin–CNT Fractionalized Nanofluid Flow in a Channel with Isothermal and Ramped Conditions

**DOI:** 10.3390/nano12081255

**Published:** 2022-04-07

**Authors:** Kashif Sadiq, Imran Siddique, Jan Awrejcewicz, Maksymilian Bednarek

**Affiliations:** 1 Department of Mathematics, University of Management and Technology, Lahore 54770, Pakistan; kashifsadiq2525@gmail.com; 2 Department of Automation, Biomechanics and Mechatronics, Lodz University of Technology, 1/15 Stefanowskiego St., 90-924 Lodz, Poland; jan.awrejcewicz@p.lodz.pl (J.A.); maksymilian.bednarek@p.lodz.pl (M.B.)

**Keywords:** nanofluid, CNTs, Soret effect, thermal radiation, ramped conditions, vertical channel

## Abstract

This article investigates heat and mass transport enrichment in natural convection fractionalized nanofluid flow inside a channel with isothermal and ramped wall conditions under the effects of chemical reactions, radiation, heat absorption, and the Soret effect. To obtain the fractional model, the Caputo time-fractional derivative definition is used, and analytical results are obtained by the Laplace transform. In two base fluids, water and glycerin, the impacts of two nanoparticles, single-wall carbon nanotubes (SWCNTs) and multiple-wall carbon nanotubes (MWCNTs), are investigated. The comparison of six distinct fluids, including water, water–SWCNT, water–MWCNT, glycerin, glycerin–SWCNT and glycerin–WMCNT, is explored graphically. Physical parameters’ effects on isothermal and ramped conditions are graphically depicted and explained in depth. For isothermal wall conditions, the variation in concentration, temperature and velocity is exponential, while for ramped wall conditions, the variation is steady. Finally, the results of skin frictions, Nusselt numbers and Sherwood numbers and for both ramped wall and isothermal wall conditions are evaluated in tabular form for various values of volume fraction. Moreover, it is observed that the temperature, velocity, Nusselt numbers and skin frictions increase by increasing the volume fraction of CNTs.

## 1. Introduction

Technology has grown rapidly in recent years in several disciplines of engineering and science, such as electronics and power production, where heat transmission is a critical phenomenon. The rapid advancement of technology necessitates new and creative heat transfer supervision. In this circumstance, advanced and creative cooling techniques must be employed. The studies attempted to produce an effective heat transport medium as heat transport managing in electronic gadgets and industries became more prominent. Several traditional strategies, such as creating a small channel and expanding the area of surface, were tried but unsuccessful owing to scientific constraints. Enhancement in nano-science, on the other hand, gives heat transfer fluids a unique quality by inserting nanoparticles in the conventional fluids. The majority of research focuses on the impact of different fluid dynamical mechanisms and flow geometries. Furthermore, the majority of past research was done using either experimental or numerical methodologies. Choi et al. [1] first discovered this phenomenon of nanoparticle dispersion in a host fluid. As a result, a large number of researchers have characterized, manufactured and tested various nanofluids in heat transfer applications [2,3]. Several experiments and numerical research revealed a new base fluid and nanoparticle combination. Carbon, semiconductors, metals, carbide ceramic, nitride ceramic and metal oxide in different forms are often employed in conventional fluids such as polymer solutions, alcohol, oil and water among the numerous varieties of nanoparticles [4]. However, due to their high thermal conductivity, carbon nanotubes (CNTs) have been found to be more effective in convective heat transfer in recent experiments. Zubair et al. [5] discussed the advantages of free convection nanofluid flow on an isothermal upright sheet by using the Crank–Nicolson numerical technique. Shahzad et al. [6] investigated the magnetohydrodynamics viscous Jeffrey nanofluid flow in a porous stretching sheet with viscous dissipation and ohmic heating. In an upright triple-tube casing, Najim et al. [7] examined the possible enhancement of circular fins on amplifying the phase-change materials’ heat response. The free convection flow of a nano-encapsulated phase-changing material suspension in an eccentric annulus was numerically investigated by Mehryan et al. [8]. Shahsavar et al. [9] used a two-phase model to discuss the entropy generation of nanofluid flow in an annulus.

Nanofluid flows are important in several engineering processes because of their widespread industrial use and high heat transfer capabilities. In recent years, a new type of nanofluid known as “hybrid nanofluids” has replaced traditional nanofluid flows to improve heat transmission. Gul et al. [10] explored the flow of a hybrid nanofluid on a stretched surface influenced by a magnetic dipole. Ramzan et al. [11] investigated the two hybrid nanofluids in a permeable medium including particles of varied shapes and water as the base fluid. In a triangular permeable cavity, Redouane et al. [12] numerically studied a water/magnesium oxide-silver hybrid nanofluid. Random motion and thermo-migration of nano particles are two properties of these items in a fluid that might impair nanofluid performance and transport. Little is known about the dynamics of water colloid mixed with three different types of nano-sized particles, with a focus on the variation of friction, mass, and heat transfer rates across the domain. Saleem et al. [13] investigated the Brownian motion and thermo-migration of a water-based ternary hybrid nanofluid on a horizontal surface. The dynamics of a ternary-hybrid nanofluid sensitive to magnetic flux density and heat source or sink on a convectively heated surface were explored by Animasaun et al. [14].

CNTs were first described in the literature by Lijima [15] in 1993. He used the Huffman and Kretschmer method to study MWCNTs. Later, in 1993, Ajayan [16] studied SWCNTs, which has a wide range of applications in electronics, healthcare, biomedicine, the environment, diesel engines, energy, and solar heaters [17]. CNT nanoparticles have significant features, such as reduced density and higher electrical and thermal conductivities when compared to standard spherical-shaped nanoparticles [18]. Aman et al. [19] examined the flow of CNT nanofluid in a channel. Using the perturbation approach, Khalid et al. [20] looked at the magneto hydrodynamic flow of a blood–CNT nanofluid in porous medium. MWCNTs and SWCNTs were considered in these investigations. They discovered that when the volume concentration grows, the temperature field increases, but the velocity field shows the reverse pattern. According to their findings, SWCNTs have a greater temperature field than MWCNTs in various based fluids [21,22,23,24] and the references therein provide some more recent work on nanofluids.

Due to the Soret effect’s applicability in engineering and science, several researchers are studying its effect on natural convection mass and heat transfer. The Soret effect is caused by temperature variations that produce mass diffusion. Soret effects are used in chemical processing, petrology, hydrology, geosciences, and isotope separation [25,26,27,28,29,30,31]. Raju et al. [32] investigated the effects of the Soret effect, magnetic force and radiation on the nanofluid flow on a moving vertical plate. RamReddy et al. [33] studied the heat and mass transport in a convective flow over a plate with the Soret effect.

Radiative heat exchange has been used in an extensive variety of applications, containing aero planes, missiles, and propulsion systems for space craft, gas turbines and nuclear plants. Furthermore, due to the vast variety of applications, mass and energy transport in the presence of chemical reactions has piqued the interest of numerous writers. Pal and Talukdar [34] studied conjugate mass and temperature transport flow via a perpendicular porous plate with chemical reaction and radiation. The influence of chemical reactions and radiation on concentration and velocity fields was discovered to be negative. Chamkha [35] conducted a numerical examination of mass and heat transmission of an electrically charged fluid across a surface with the effect of chemical reaction. Uwanta and Omokhuale [36] investigated chemical reactions and radiation effects on a thick fluid in a plane. The Laplace transform approach was used by Das et al. [37] to develop analytical results to the unsteady problem of natural convection flow and mass diffusion on an upright plate with the heat radiation effect. Several steps have been taken to inspect the outcomes of radiation and chemical reaction in a variety of physical settings [38,39,40,41,42,43].

Fluid flow and coupled mass and heat transmission across a channel have attained less consideration than a single plate. This design may be found in a variety of applications, including fire engineering, petroleum reservoirs, nuclear energy, and combustion modeling and so on. Many engineering systems exhibit transport phenomena combining the combined impact of concentration and thermal buoyancy. They are often found in modern thermal protection devices, chemical distilleries, building ventilation systems, solar panels, heat exchangers and electric circuits [44,45,46,47]. The majority of research focuses on the impact of different fluid dynamical mechanisms and flow geometry. Furthermore, the majority of past research was done using either experimental or numerical methodologies.

According to the literature review, there is a research gap in the area of mass and heat transport examination on the convection of water/glycerin–CNT nanofluids owing to ramped boundary conditions. As a result, the goal of this study is simple and novel: to investigate the influence of carbon nanotubes, thermal radiation, heat absorption and the Soret effect on water/glycerin nanofluid flow under ramped and isothermal conditions in a channel. These are vital in medical instruments, food processing, crystal growing operations, geothermal and geophysical systems, storage devices, and aerospace engineering to aid in the understanding of heat transport and fluid flow. Here we study the natural convection fractionalized nanofluid flows within a channel with isothermal and ramped wall conditions under the effect of Soret, chemical reactions, heat absorption and radiation. The Caputo time fractional models are solved by the Laplace transform. The effects of two nanoparticles, SWCNTs and MWCNTs, are examined in two base fluids, water and glycerin. The comparison of six different nanofluids is investigated and graphed, including water, water–SWCNT, water–MWCNT, glycerin, glycerin–SWCNT and glycerin–WMCNT. The impacts of physical factors on isothermal and ramped conditions are graphically displayed and thoroughly discussed.

## 2. Mathematical Model

Consider an unsteady natural convection flow of nanofluid inside two infinite upright plates in the existence of chemical reaction, heat absorption, Soret effect and radiation with ramped conditions in $\tilde{x_{1}},\;\;\tilde{y_{1}}$ plane, where the left wall is along $\tilde{x_{1}}$-axis.

**Figure 1:**
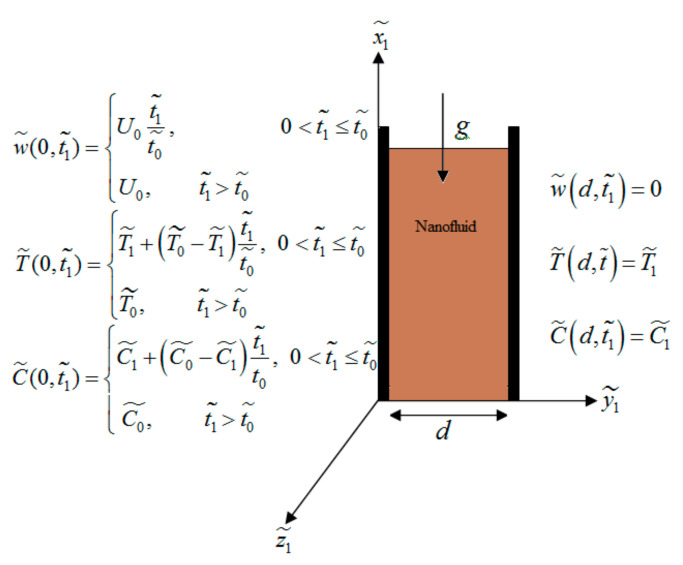
Flow geometry.

Initially, at $\tilde{t_{1}}=0$ ($\tilde{y_{1}}=0$ and $\tilde{y_{1}}=d$), the temperature, velocity and concentration are uniformed. At $0<\tilde{t}<\tilde{t_{0}},$ the temperature, velocity and concentration of the left wall varies temporarily to $\tilde{T_{1}}+\left(\tilde{T_{0}}-\tilde{T_{1}}\right)\frac{\tilde{t_{1}}}{\tilde{t_{0}}}$, $U_{0}\frac{\tilde{t_{1}}}{\tilde{t_{0}}}$ and $\tilde{C_{1}}+\left(\tilde{C_{0}}-\tilde{C_{1}}\right)\frac{\tilde{t_{1}}}{\tilde{t_{0}}}$, respectively. After $\tilde{t_{1}}>\tilde{t_{0}},$ the system regains its original position (see Figure 1). Thermo-physical features of CNTs and fluids are assumed constant and shown in Table 1.

The governing equations are [49]:

(1) $$\rho_{nf}\frac{\partial \tilde{w}(\tilde{y_{1}},\tilde{t_{1}})}{\partial \tilde{t_{1}}}=\mu_{nf}\frac{\partial^{2}\tilde{w}(\tilde{y_{1}},\tilde{t_{1}})}{\partial {\tilde{y_{1}}}^{2}}+g{\left(\rho \beta_{T}\right)}_{nf}\left(\tilde{T}(\tilde{y_{1}},\tilde{t_{1}})-\tilde{T_{1}}\right)+g{\left(\rho \beta_{C}\right)}_{nf}\left(\tilde{C}(\tilde{y_{1}},\tilde{t_{1}})-\tilde{C_{1}}\right),$$

(2) $${\left(\rho c_{p}\right)}_{nf}\frac{\partial \tilde{T}(\tilde{y_{1}},\tilde{t_{1}})}{\partial \tilde{t_{1}}}=k_{nf}\frac{\partial^{2}\tilde{T}(\tilde{y_{1}},\tilde{t_{1}})}{\partial {\tilde{y_{1}}}^{2}}-\frac{\partial q_{r}}{\partial y_{1}}-Q_{0}\left(\tilde{T}(\tilde{y_{1}},\tilde{t_{1}})-\tilde{T_{1}}\right),$$

(3) $$\frac{\partial \tilde{C}(\tilde{y_{1}},\tilde{t_{1}})}{\partial \tilde{t_{1}}}=D_{nf}\frac{\partial^{2}\tilde{C}(\tilde{y_{1}},\tilde{t_{1}})}{\partial {\tilde{y_{1}}}^{2}}+\frac{D_{nf}k_{T}}{T_{m}}\frac{\partial^{2}\tilde{T}(\tilde{y_{1}},\tilde{t_{1}})}{\partial {\tilde{y_{1}}}^{2}}-R_{0}\left(\tilde{C}(\tilde{y_{1}},\tilde{t_{1}})-\tilde{C_{1}}\right),$$

with related conditions

(4) $$\tilde{w}(\tilde{y_{1}},0)=0,\;\;\tilde{T}(\tilde{y_{1}},0)=\tilde{T_{1}},\;\;\;\tilde{C}(\tilde{y_{1}},0)=\tilde{C_{1}},\;\;\;\;\;\;\;\;\;\;\;\;0\leq \tilde{y_{1}}\leq d,$$

(5) $$\begin{array}{l} \tilde{w}\left(0,\tilde{t_{1}}\right)=\left\{\begin{array}{l} U_{0}\frac{\tilde{t_{1}}}{\tilde{t_{0}}},0<\tilde{t_{1}}\leq \tilde{t_{0}};\\ U_{0},\;\;\tilde{t_{1}}>\tilde{t_{0}}, \end{array}\right.,\;\tilde{T}\left(0,\tilde{t}\right)=\left\{\begin{array}{l} \tilde{T_{1}}+\left(\tilde{T_{0}}-\tilde{T_{1}}\right)\frac{\tilde{t_{1}}}{\tilde{t_{0}}},0<\tilde{t_{1}}\leq \tilde{t_{0}};\\ \tilde{T_{0}},\;\;\tilde{t_{1}}>\tilde{t_{0}}, \end{array}\right.,\\ \tilde{C}\left(0,\tilde{t_{1}}\right)=\left\{\begin{array}{l} \tilde{C_{1}}+\left(\tilde{C_{0}}-\tilde{C_{1}}\right)\frac{\tilde{t_{1}}}{\tilde{t_{0}}},\;0<\tilde{t_{1}}\leq \tilde{t_{0}};\\ \tilde{C_{0}},\;\;\tilde{t_{1}}>\tilde{t_{0}}, \end{array}\right., \end{array}$$

(6) $$\tilde{w}\left(d,\tilde{t_{1}}\right)=0,\;\;\tilde{T}\left(d,\tilde{t_{1}}\right)=\tilde{T_{1}},\;\;\tilde{C}\left(d,\tilde{t_{1}}\right)=\tilde{C_{1}}.$$

The characteristics of nanofluid are defined by [50,51,52,53].

(7) $$\mu_{nf}=\mu_{f}{\left(1-\varphi \right)}^{-2.5},\;\;\frac{{\left(\rho c_{p}\right)}_{nf}}{{\left(\rho c_{p}\right)}_{f}}-(1-\varphi )=\varphi \frac{{\left(\rho c_{p}\right)}_{s}}{{\left(\rho c_{p}\right)}_{f}},\;\;\frac{\rho_{nf}}{\rho_{f}}-(1-\varphi )=\varphi \frac{\rho_{s}}{\rho_{f}},$$

(8) $$\frac{{\left(\rho \beta_{C}\right)}_{nf}}{{\left(\rho \beta_{C}\right)}_{f}}-(1-\varphi )=\varphi \frac{{\left(\rho \beta_{C}\right)}_{CNT}}{{\left(\rho \beta_{C}\right)}_{f}},\;\;\frac{{\left(\rho \beta_{T}\right)}_{nf}}{{\left(\rho \beta_{T}\right)}_{f}}-(1-\varphi )=\varphi \frac{{\left(\rho \beta_{T}\right)}_{CNT}}{{\left(\rho \beta_{T}\right)}_{f}},$$

(9) $$D_{nf}=\left(1-\varphi \right)D_{f},\;\;k_{nf}=\frac{2\varphi \left(\frac{k_{CNT}}{k_{CNT}-k_{f}}\right)\ln\frac{k_{CNT}+k_{f}}{2k_{f}}+1-\varphi }{2\varphi \left(\frac{k_{f}}{k_{CNT}-k_{f}}\right)\ln\frac{k_{CNT}+k_{f}}{2k_{f}}+1-\varphi }k_{f}.$$

Placing the following non-dimensional parameters

(10) $$\begin{array}{c} u=\frac{\tilde{w}}{U_{0}},\;t=\frac{\tilde{t_{1}}}{\tilde{t_{0}}},\;\tilde{t_{0}}=\frac{d^{2}}{v_{f}},\;\;y=\frac{\tilde{y_{1}}}{d},\;\;T=\frac{\tilde{T}-\tilde{T_{1}}}{\tilde{T_{0}}-\tilde{T_{1}}},\;C=\frac{\tilde{C}-\tilde{C_{1}}}{\tilde{C_{0}}-\tilde{C_{1}}},\;\;a_{0}={\left(1-\varphi \right)}^{-2.5}\frac{\rho_{f}}{\rho_{nf}},a_{1}=G_{r}\frac{{\left(\beta_{T}\right)}_{nf}}{{\left(\beta_{T}\right)}_{f}},\\ a_{2}=G_{m}\frac{{\left(\beta_{C}\right)}_{nf}}{{\left(\beta_{C}\right)}_{f}},a_{3}=\frac{1}{\mathrm{Pr}}\left(\frac{k_{nf}}{k_{f}}+Nr\right)\frac{{\left(\rho c_{p}\right)}_{f}}{{\left(\rho c_{p}\right)}_{nf}},a_{4}=Q\frac{{\left(\rho c_{p}\right)}_{f}}{{\left(\rho c_{p}\right)}_{nf}},a_{5}=\frac{1-\varphi }{Sc},a_{6}=Sr\left(1-\varphi \right),\\ R=\frac{R_{0}d^{2}}{v_{f}},G_{m}=\frac{g{\left(\beta_{C}\right)}_{f}(C_{0}-C_{1})d^{2}}{U_{0}v_{f}},Sc=\frac{v_{f}}{D_{f}},G_{r}=\frac{g{\left(\beta_{T}\right)}_{f}(T_{0}-T_{1})d^{2}}{U_{0}v_{f}},\;\mathrm{Pr}=\frac{{\left(\mu c_{p}\right)}_{f}}{k_{f}},\\ Nr=\frac{16\sigma_{1}T_{1}{}^{3}}{3k_{1}k_{f}},Q=\frac{Q_{0}d^{2}}{{\left(\rho c_{p}\right)}_{f}v_{f}},Sr=\frac{D_{f}k_{T}\left(T_{0}-T_{1}\right)}{T_{m}v_{f}\left(C_{0}-C_{1}\right)}, \end{array}$$

into Equations (1)–(6), we get:

(11) $$\frac{\partial u(y,t)}{\partial t}=a_{0}\frac{\partial^{2}u(y,t)}{\partial y^{2}}+a_{1}\theta (y,t)+a_{2}C(y,t),$$

(12) $$\frac{\partial \theta (y,t)}{\partial t}=a_{3}\frac{\partial^{2}\theta (y,t)}{\partial y^{2}}-a_{4}\theta (y,t),$$

(13) $$\frac{\partial C(y,t)}{\partial t}=a_{5}\frac{\partial^{2}C(y,t)}{\partial y^{2}}+a_{6}\frac{\partial^{2}\theta (y,t)}{\partial y^{2}}-RC(y,t),$$

(14) C(y,0)=u(y,0)=θ(y,0)=0;            0≤y≤1,

(15) $$C\left(0,t\right)=u(0,t)=\theta (0,t)=\left\{\begin{array}{c} t,\;\;\;\;\;\;\;\;\;\;\;\;0<t\leq 1;\\ 1,\;\;\;\;\;\;\;\;\;\;\;\;\;\;\;\;\;\;\;t>1 \end{array}\right.,$$

(16)  C(1,t)=u(1,t)= θ(1,t)=0.

Caputo time-fractional derivative is used in Equations (11)–(13) to have fractional models:

(17) $$D_{t}{}^{\alpha }u\left(y,t\right)=a_{0}\frac{\partial^{2}u(y,t)}{\partial y^{2}}+a_{1}\theta (y,t)+a_{2}C(y,t),$$

(18) $$D_{t}{}^{\alpha }\theta \left(y,t\right)=a_{3}\frac{\partial^{2}\theta (y,t)}{\partial y^{2}}-a_{4}\theta (y,t),$$

(19) $$D_{t}{}^{\alpha }C\left(y,t\right)=a_{5}\frac{\partial^{2}C(y,t)}{\partial y^{2}}+a_{6}\frac{\partial^{2}\theta (y,t)}{\partial y^{2}}-RC(y,t).$$

## 3. Solution of the Problem

Applying the Laplace transform to Equations (15)–(19), (6) and using (14), we obtain:

(20) $$a_{0}\;\frac{\partial^{2}\bar{u}(y,s)}{\partial y^{2}}-s^{\alpha }\bar{u}(y,s)+a_{1}\bar{\theta }(y,s)+a_{2}\bar{C}(y,s)=0,$$

(21) $$a_{3}\;\frac{\partial^{2}\bar{\theta }(y,s)}{\partial y^{2}}-\left(s^{\alpha }+a_{4}\right)\bar{\theta }(y,s)=0,$$

(22) $$a_{5}\;\frac{\partial^{2}\bar{C}(y,s)}{\partial y^{2}}-\left(s^{\alpha }+R\right)\bar{C}(y,s)+a_{6}\frac{\partial^{2}\bar{\theta }(y,s)}{\partial y^{2}}=0,$$

(23) $$\bar{C}(0,s)=\bar{u}(0,s)=\bar{\theta }(0,s)=\left(1-e^{-s}\right)s^{-2},$$

(24) $$\bar{C}(1,s)=\bar{u}(1,s)=\bar{\theta }(1,s)=0.$$

The solutions of Equations (20)–(22) by using Equations (23) and (24) are:

(25) $$\begin{array}{c} \bar{u}(y,s)=\left(1-e^{-s}\right)\frac{\sinh\left[\left(1-y\right)\sqrt{\frac{s^{\alpha }}{a_{0}}}\right]}{s^{2}\sinh\left[\sqrt{\frac{s^{\alpha }}{a_{0}}}\right]}\\ +\frac{b_{2}\left(1-e^{-s}\right)}{s^{\alpha }+b_{3}}\left(1+\frac{b_{0}\left(s^{\alpha }+a_{4}\right)}{s^{\alpha }+b_{1}}\right)\left[\frac{\sinh\left[\left(1-y\right)\sqrt{\frac{s^{\alpha }}{a_{0}}}\right]}{s^{2}\sinh\left[\sqrt{\frac{s^{\alpha }}{a_{0}}}\right]}-\frac{\sinh\left[\left(1-y\right)\sqrt{\frac{s^{\alpha }+R}{a_{5}}}\right]}{s^{2}\sinh\left[\sqrt{\frac{s^{\alpha }+R}{a_{5}}}\right]}\right]\\ +\frac{b_{4}\left(1-e^{-s}\right)}{s^{\alpha }+b_{5}}\left(a_{1}-\frac{a_{2}b_{0}\left(s^{\alpha }+a_{4}\right)}{s^{\alpha }+b_{1}}\right)\left[\frac{\sinh\left[\left(1-y\right)\sqrt{\frac{s^{\alpha }}{a_{0}}}\right]}{s^{2}\sinh\left[\sqrt{\frac{s^{\alpha }}{a_{0}}}\right]}-\frac{\sinh\left[\left(1-y\right)\sqrt{\frac{s^{\alpha }+a_{4}}{a_{3}}}\right]}{s^{2}\sinh\left[\sqrt{\frac{s^{\alpha }+a_{4}}{a_{3}}}\right]}\right] \end{array}$$

(26) $$\bar{\theta }(y,s)=\left(1-e^{-s}\right)\frac{\sinh\left[\left(1-y\right)\sqrt{\frac{s^{\alpha }+a_{4}}{a_{3}}}\right]}{s^{2}\sinh\left[\sqrt{\frac{s^{\alpha }+a_{4}}{a_{3}}}\right]},$$

(27) $$\begin{array}{l} \bar{C}(y,s)=\left(1-e^{-s}\right)\frac{\sinh\left[\left(1-y\right)\sqrt{\frac{s^{\alpha }+R}{a_{5}}}\right]}{s^{2}\sinh\left[\sqrt{\frac{s^{\alpha }+R}{a_{5}}}\right]}\\ +b_{0}\left(1-e^{-s}\right)\left[1+\frac{a_{4}-b_{1}}{s^{\alpha }+b_{1}}\right]\left[\frac{\sinh\left[\left(1-y\right)\sqrt{\frac{s^{\alpha }+R}{a_{5}}}\right]}{s^{2}\sinh\left[\sqrt{\frac{s^{\alpha }+R}{a_{5}}}\right]}-\frac{\sinh\left[\left(1-y\right)\sqrt{\frac{s^{\alpha }+a_{4}}{a_{3}}}\right]}{s^{2}\sinh\left[\sqrt{\frac{s^{\alpha }+a_{4}}{a_{3}}}\right]}\right], \end{array}$$

where

(28) $$b_{0}=\frac{a_{6}}{a_{5}-a_{3}},b_{1}=\frac{a_{4}a_{5}-a_{3}R}{a_{5}-a_{3}},b_{2}=\frac{a_{2}a_{5}}{a_{0}-a_{5}},b_{3}=\frac{a_{0}R}{a_{0}-a_{5}},b_{4}=\frac{a_{3}}{a_{0}-a_{3}},b_{5}=\frac{a_{0}a_{4}}{a_{0}-a_{3}}.$$

The inverse Laplace transform of Equations (25)–(27) gives:

(29) $$u(y,t)=u\prime \left(y,t\right)-H\left(t-1\right)u\prime \left(y,t-1\right),$$

(30) $$\theta (y,t)=\theta \prime \left(y,t\right)-H\left(t-1\right)\theta \prime \left(y,t-1\right),$$

(31) $$C(y,t)=C\prime \left(y,t\right)-H\left(t-1\right)C\prime \left(y,t-1\right),$$

where

(32) $$\begin{array}{ll} u\prime \left(y,t\right) & =\int_{0}^{t}\frac{{\left(t-\tau \right)}^{1-\alpha }}{\Gamma \left(2-\alpha \right)}f\left(\frac{1-y}{\sqrt{a_{0}}},\tau ,0,\frac{1}{\sqrt{a_{0}}}\right)d\tau \\ & +b_{2}\left[\left(1-\frac{b_{0}\left(a_{4}-b_{3}\right)}{b_{1}-b_{3}}\right)g_{2}\left(t\right)+\frac{b_{0}\left(a_{4}-b_{1}\right)}{b_{1}-b_{3}}g_{1}\left(t\right)\right]\\ & \;*\left[\int_{0}^{t}\frac{{\left(t-\tau \right)}^{-\alpha }}{\Gamma \left(1-\alpha \right)}f\left(\frac{1-y}{\sqrt{a_{0}}},\tau ,0,\frac{1}{\sqrt{a_{0}}}\right)d\tau \right.-\left.\int_{0}^{t}\frac{{\left(t-\tau \right)}^{-\alpha }}{\Gamma \left(1-\alpha \right)}f\left(\frac{1-y}{\sqrt{a_{5}}},\tau ,R,\frac{1}{\sqrt{a_{5}}}\right)d\tau \right]\\ & \;+b_{4}\left[\left(a_{1}-\frac{a_{2}b_{0}\left(a_{4}-b_{5}\right)}{b_{1}-b_{5}}\right)g_{3}\left(t\right)+\frac{a_{2}b_{0}\left(a_{4}-b_{1}\right)}{b_{1}-b_{5}}g_{1}\left(t\right)\right]\\ & \;*\left[\int_{0}^{t}\frac{{\left(t-\tau \right)}^{-\alpha }}{\Gamma \left(1-\alpha \right)}f\left(\frac{1-y}{\sqrt{a_{0}}},\tau ,0,\frac{1}{\sqrt{a_{0}}}\right)d\tau \right.-\left.\int_{0}^{t}\frac{{\left(t-\tau \right)}^{-\alpha }}{\Gamma \left(1-\alpha \right)}f\left(\frac{1-y}{\sqrt{a_{3}}},\tau ,a_{4},\frac{1}{\sqrt{a_{3}}}\right)d\tau \right], \end{array}$$

(33) $$\theta \prime (y,t)=\sum_{n=0}^{\infty }\int_{0}^{t}\frac{{\left(t-\tau \right)}^{-\alpha }}{\Gamma \left(1-\alpha \right)}f\left(\frac{1-y}{\sqrt{a_{3}}},\tau ,a_{4},\frac{1}{\sqrt{a_{3}}}\right)d\tau ,$$

(34) $$\begin{array}{l} C\prime (y,t)=\int_{0}^{t}\frac{{\left(t-\tau \right)}^{1-\alpha }}{\Gamma \left(2-\alpha \right)}f\left(\frac{1-y}{\sqrt{a_{5}}},\tau ,R,\frac{1}{\sqrt{a_{5}}}\right)d\tau \\ \;\;\;\;\;\;\;\;\;\;\;\;\;\;\;\;\;\;\;\;+b_{0}\left[1+\left(a_{4}-b_{1}\right)g_{1}\left(t\right)\right]*\left[\int_{0}^{t}\frac{{\left(t-\tau \right)}^{-\alpha }}{\Gamma \left(1-\alpha \right)}f\left(\frac{1-y}{\sqrt{a_{5}}},\tau ,R,\frac{1}{\sqrt{a_{5}}}\right)d\tau \right.\\ \;\;\;\;\;\;\;\;\;\;\;\;\;\;\;\;\;\;\;\;-\left.\int_{0}^{t}\frac{{\left(t-\tau \right)}^{-\alpha }}{\Gamma \left(1-\alpha \right)}f\left(\frac{1-y}{\sqrt{a_{3}}},\tau ,a_{4},\frac{1}{\sqrt{a_{3}}}\right)d\tau \right], \end{array}$$

and

(35) $$F\left(x,s^{\alpha },y,z\right)=\frac{\sinh(x\sqrt{s^{\alpha }+y})}{s^{\alpha }\sinh(z\sqrt{s^{\alpha }+y})}=\sum_{k=0}^{\infty }\left[\frac{e^{-\left[\left(2k+1\right)z-x\right]\sqrt{s^{\alpha }+y}}}{s^{\alpha }}-\frac{e^{-\left[\left(2n+1\right)z+x\right]\sqrt{s^{\alpha }+y}}}{s^{\alpha }}\right],$$

(36) $$f\left(x,t,y,z\right)=L^{-1}\left\{F\left(x,s^{\alpha },y,z\right)\right\}=\left\{\begin{array}{c} \int_{0}^{\infty }f\left(x,u,y,z\right)t^{-1}\phi \left(0,-\alpha ,-ut^{-\alpha }\right)du,0<\alpha <1\\ f\left(x,t,x,z\right),\;\;\;\;\;\;\;\;\;\;\;\;\;\;\;\;\;\;\;\;\;\;\;\;\;\;\;\;\;\;\;\;\;\;\;\;\;\;\;\;\;\;\;\;\alpha =1 \end{array}\right.,$$

(37) $$F\left(x,s,y,z\right)=\frac{\sinh(x\sqrt{s+y})}{s^{\alpha }\sinh(z\sqrt{s+y})}=\sum_{k=0}^{\infty }\left[\frac{e^{-\left[\left(2k+1\right)z-x\right]\sqrt{s+y}}}{s}-\frac{e^{-\left[\left(2n+1\right)z+x\right]\sqrt{s+y}}}{s}\right],$$

(38) $$f\left(x,t,y,z\right)=L^{-1}\left\{F\left(x,s,y,z\right)\right\}=\sum_{k=0}^{\infty }\left[\Psi \left(x,t,y,z\right)-\Psi \left(-x,t,y,z\right)\right],$$

(39) $$\begin{array}{l} \Psi \left(x,t,y,z\right)=\frac{1}{2}\left[e^{-\left[\left(2k+1\right)z-x\right]\sqrt{y}}erfc\left(\frac{\left(2k+1\right)z-x}{2\sqrt{t}}-\sqrt{yt}\right)\right.\\ \left.\;\;\;\;\;\;\;\;\;\;\;\;\;\;\;\;\;\;\;\;\;\;\;\;\;\;\;\;\;+e^{\left[\left(2k+1\right)z-x\right]\sqrt{y}}erfc\left(\frac{\left(2k+1\right)z-x}{2\sqrt{t}}+\sqrt{yt}\right)\right], \end{array}$$

(40) $$g_{1}\left(t\right)=L^{-1}\left\{\frac{1}{s\left(s^{\alpha }+b_{1}\right)}\right\}=\int_{0}^{t}\tau^{\alpha -1}E_{\alpha ,\alpha }\left(-b_{1}\tau^{\alpha }\right)d\tau ,$$

(41) $$g_{2}\left(t\right)=L^{-1}\left\{\frac{1}{s\left(s^{\alpha }+b_{3}\right)}\right\}=\int_{0}^{t}\tau^{\alpha -1}E_{\alpha ,\alpha }\left(-b_{3}\tau^{\alpha }\right)d\tau ,$$

(42) $$g_{3}\left(t\right)=L^{-1}\left\{\frac{1}{s\left(s^{\alpha }+b_{3}\right)}\right\}=\int_{0}^{t}\tau^{\alpha -1}E_{\alpha ,\alpha }\left(-b_{5}\tau^{\alpha }\right)d\tau .$$

## 4. Nusselt Numbers, Skin Frictions and Sherwood Numbers

The Sherwood numbers, skin friction and Nusselt numbers on both walls of the channel can express as [54]:

(43) $$\begin{array}{l} skin\;frictions=Sk_{0,1}=\;-\frac{\mu_{nf}}{\mu_{f}}L^{-1}{\left\{\frac{\partial \bar{u}\left(y,s\right)}{\partial y}\right\}}_{y=0,1},\;\\ Nusselt\;numbers=Nu_{0,1}=-\frac{k_{nf}}{k_{f}}L^{-1}{\left\{\frac{\partial \bar{\theta }\left(y,s\right)}{\partial y}\right\}}_{y=0,1},\\ Sherwood\;numbers=Sh_{0,1}=\;-\frac{D_{nf}}{D_{f}}L^{-1}{\left\{\frac{\partial \bar{C}\left(y,s\right)}{\partial y}\right\}}_{y=0,1}. \end{array}$$

## 5. Graphical Results and Discussions

**Figure 2:**
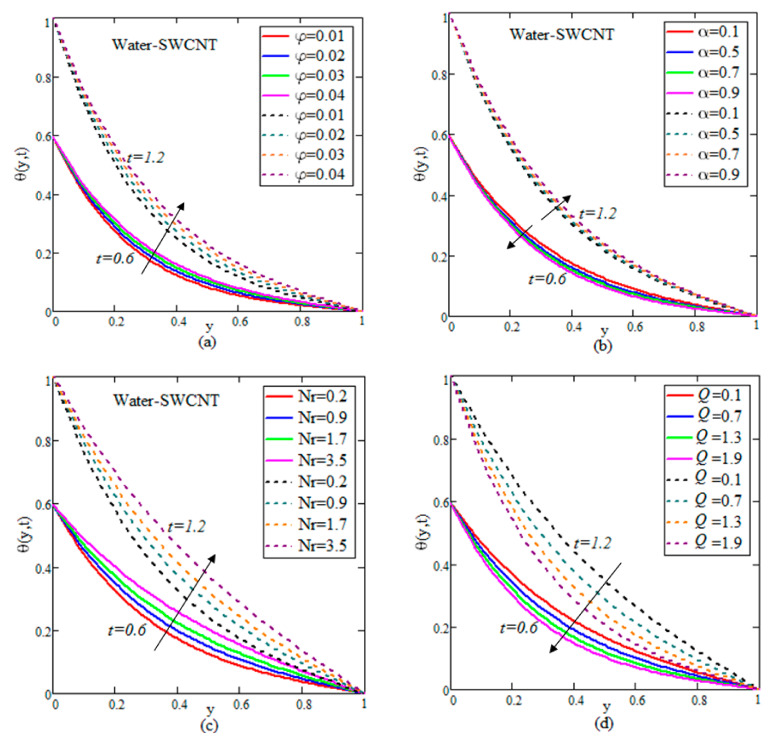
(**a**–**d**) Deviation of temperatures.

**Figure 3:**
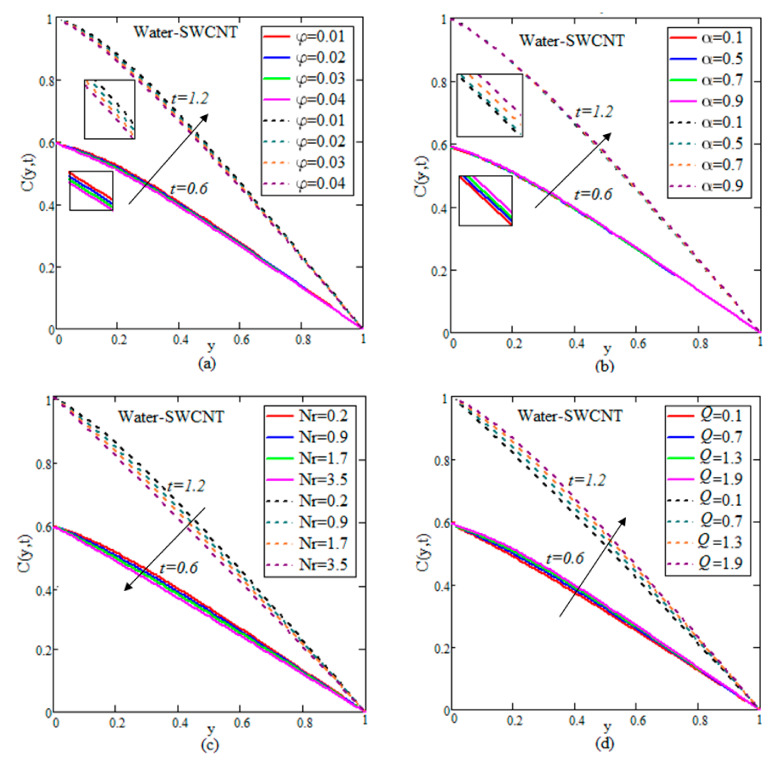

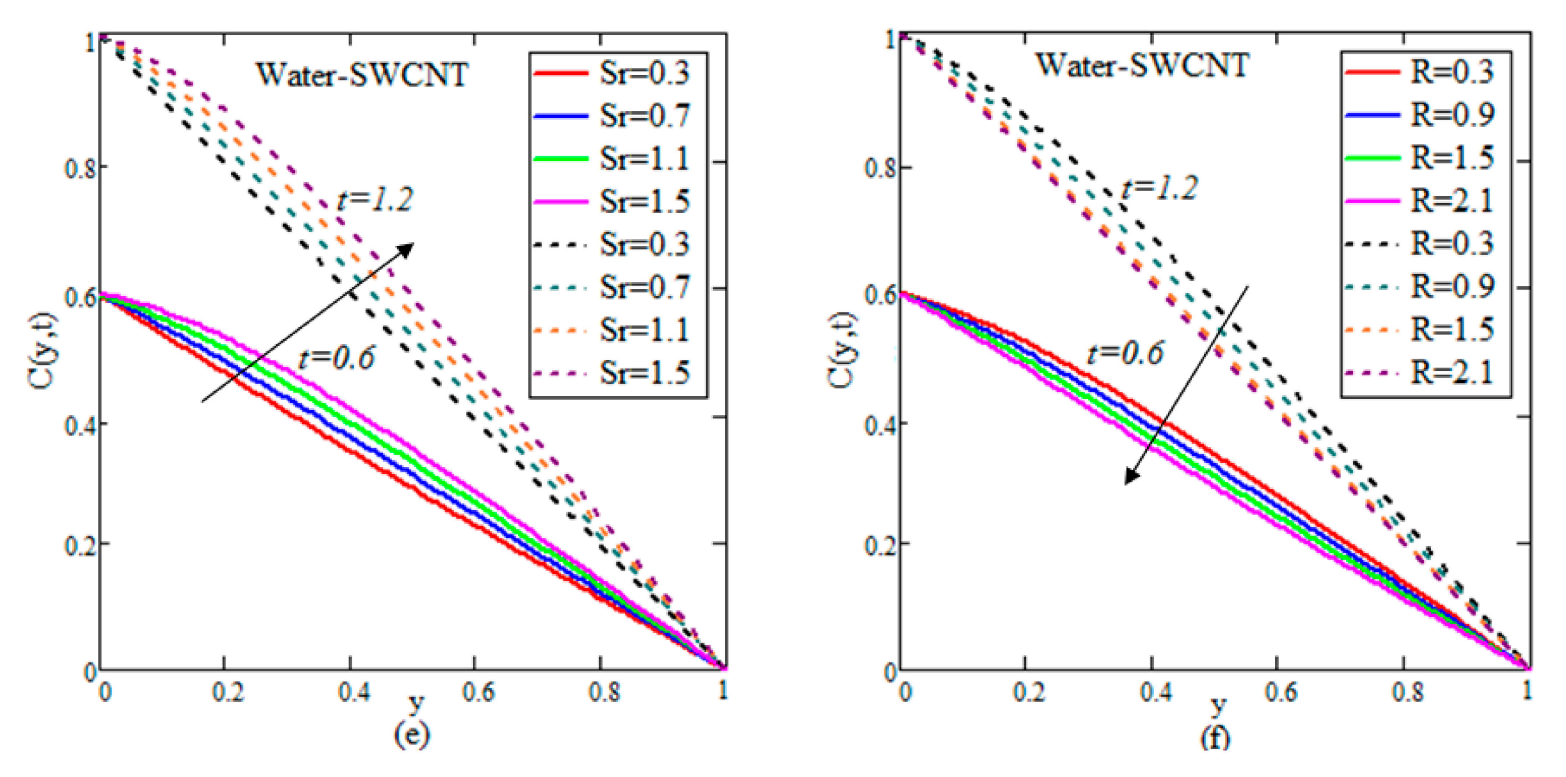
(**a**–**f**) Deviation of concentrations.

**Figure 4:**
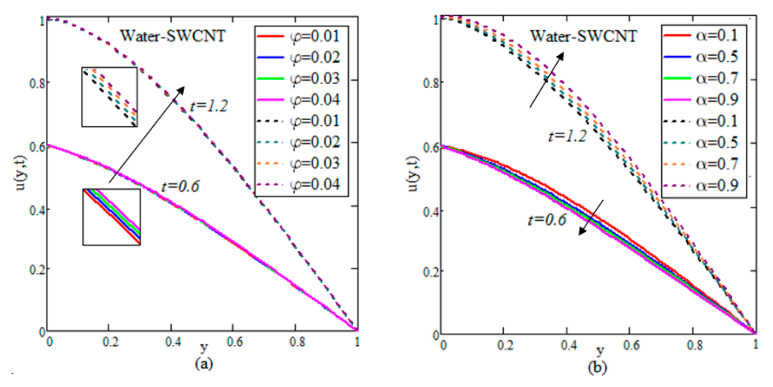

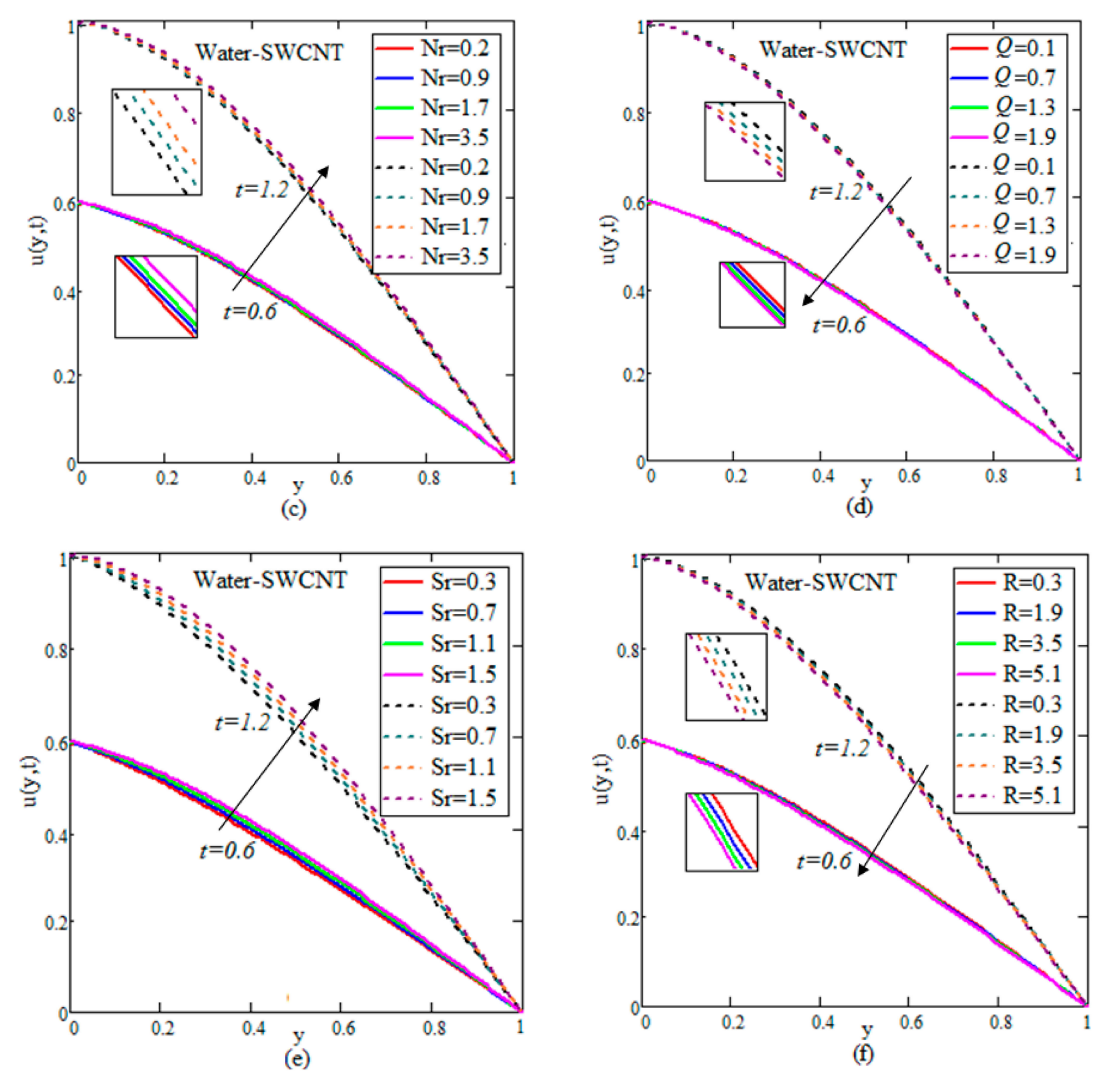
(**a**–**f**) Deviation of velocities.

**Figure 5:**
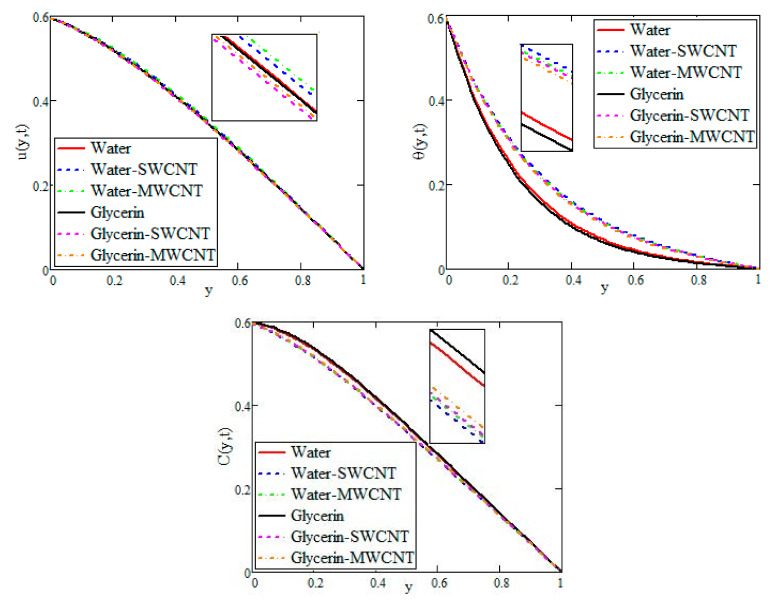
Comparison of velocities, temperatures and concentrations at *t =* 0.6.

**Figure 6:**
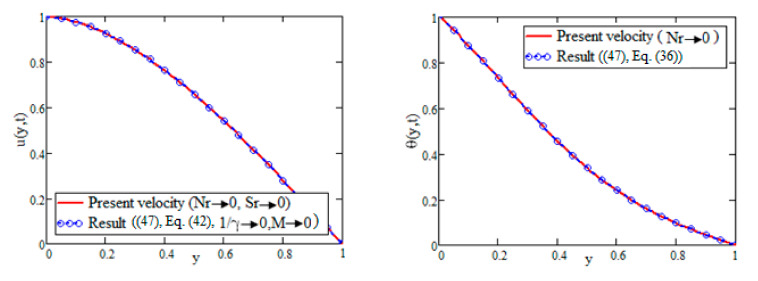

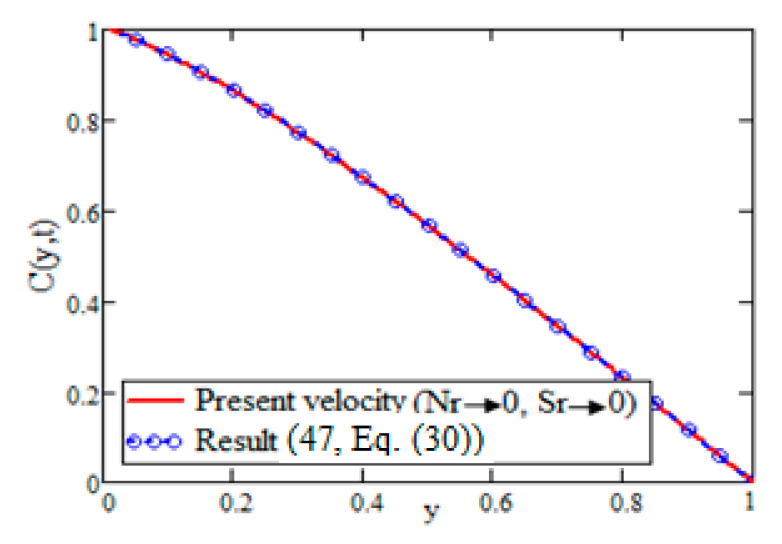
Comparison of results at *t =* 1.2.

The results for velocity, concentration and temperature profiles are calculated for diverse flow restrictions such as φ, α, Sr, Nr, R and *Q*. The velocity, concentration and temperature fields are graphically shown in Figure 2, Figure 3, Figure 4, Figure 5 and Figure 6 to investigate the physical elements of the problem. The impact of φ on the rate of change of flow, mass and temperature are shown in Table 2, Table 3 and Table 4. It is worth noting that the ramped and isothermal boundary conditions are set at t=0.6 and t=1.2, respectively. For the entire discussion of graphs and tables, we used φ=0.04, Q=1.5, Nr=0.05, Sc=0.3, α=0.5, $G_{r}=1.5,$ R=0.9, Sr=1.2 and $G_{m}=2.2,$ excluding the deviation in relevant figures. Figure 2, Figure 3 and Figure 4 show the variation of water–SWCNT.

Figure 2a illustrates the influence of φ on the temperature. The temperature field rises with raising the values of φ due to SWCNTs’ high effective heat conductivity. When φ increases, the heat conductivity increases, and this raises the temperature. The impact of volume fraction on the concentration of fluid is shown in Figure 3a. The concentration increases due to the rise in temperature influenced by the Soret effect, which reduces the thickness of the fluid. In Figure 4a, the influence of φ on the velocity field is illustrated. Because of the effective density of SWCNTs, the velocity reduces as the φ increases. An increase in φ causes a rise in the thickness of the nanofluid, which causes the fluid velocity to slow down. 

The control of α on the temperature is shown in Figure 2b for both isothermal and ramped wall temperatures. In the instance of ramped temperature, the temperature releases as the value of α increases. However, the temperature rises in the case of an isothermal instance. This pattern is shifting away from the plate. The temperature field’s trend may be physically rationalized, since a rise in α produces a decline in the boundary layer, resulting in a boost in the temperature field. Figure 3b illustrates the increase in temperature by rising in concentration. The impact of fractional parameters on the velocity is shown in Figure 4b. For growing values of α, the velocity reduces with ramped conditions and increases with the isothermal temperatures.

Figure 2c, Figure 3c and Figure 4c show how radiation parameters affect the temperature, concentration and velocity fields. The concentration of the flow decreases by increasing the values of *Nr*, while the temperature and velocity of the flow increases by increasing the value of *Nr*. The heat transmitted to the fluid rises as well, boosting the fluid’s temperature and enhancing fractional nanofluid flow.

Figure 2d, Figure 3d and Figure 4d demonstrate the importance of heat suction in the temperature, concentration and velocity profiles. Heat suction is denoted by the positive *Q* values. Figure 2d depicts the decrease by raising the value of *Q*. Rises *Q*, causing the decreasing of temperature, as shown in the graph. Furthermore, as compared to the ramped wall temperature, the solution has an impressive profile, when the wall temperature is constant. For higher values of *Q*, more energy is absorbed, which increases the concentration and decreases the velocity. The influence of *Sr* on concentration and velocity is depicted in Figure 3e and Figure 4e. The velocity and concentration fields of the flow increase as *Sr* increases. The impact of R is demonstrated in Figure 3f and Figure 4f. A decline is observed in both the mass and flow of nanofluid under increasing chemical reaction.

Figure 5 illustrates the comparison of water, water–SWCNT, water–MWCNT, glycerin, glycerin–SWCNT and glycerin–MWCNT velocities, temperatures and concentrations under the ramped boundary conditions. For ramped conditions, water–MWCNT has the maximum velocity, while glycerin–SWCNT has the lowest velocity. Because of the increase in thermal conductivity, the temperature profile of water–SWCNT is higher than the other fluids. Glycerin–MWCNT has a greater concentration than the other fluids under study.

Figure 6 shows that our obtained results are identical to those obtained in [47], in the absence of *Sr*, Nr, Casson parameter (γ) and magnetic field (M) at *t* = 1.2.

The fluctuation of skin friction with volume fraction can be seen in Table 2. Skin frictions of water–SWCNT, water–MWCNT, glycerin–SWCNT and glycerin–WMCNT increase by increasing *φ* for both isothermal and ramped conditions on both walls of the channel.

Table 3 shows the diverse values of Nusselt numbers, which measure the heat transmission rate. The rate of heat transmission rises with the rise of CNT volume fraction in two fluids (water and glycerin). Because the Nusselt number is the proportion of convection to conduction, it follows that convection grows as *φ*. The heat conductivity of the nanofluids increases by inserting more CNTs, and thus the heat transfer increases.

Table 4 shows the variation of Sherwood numbers due to increasing values of *φ*. Sherwood numbers of water–SWCNT, water–MWCNT, glycerin–SWCNT and glycerin–WMCNT increases at *y* = 0 and decreases at *y* = 1 by increasing *φ* for both isothermal and ramped conditions on both walls of the channel.

## 6. Conclusions

Natural convection nanofluid flow through a perpendicular channel with isothermal and ramped wall conditions is studied in this work. The problem also takes into account the Soret effect, chemical reactions, heat absorption and radiation effects. The Laplace transform technique is used to solve the Caputo time-fractional models. In two base fluids, water and glycerin, the impacts of two nanoparticles, SWCNTs and MWCNTs, are investigated. The comparison of six distinct fluids, including water, water–SWCNT, water–MWCNT, glycerin, glycerin–SWCNT and glycerin–WMCNT, is explored graphically. Physical parameter effects on isothermal and ramped conditions are visually depicted and explained in depth. We observed that ramped conditions can regulate flow, mass and energy. For isothermal conditions, the variation in concentration, velocity and temperature is exponential, while for ramped wall conditions, the variation is steady. Finally, the results of skin frictions, Nusselt numbers and Sherwood numbers on both channel walls (*y* = 0 and *y* = 1) for ramped wall and isothermal wall conditions are shown. The significant results for velocity, concentration and temperature are graphically highlighted and discussed in detail.

The significant outcomes of this study are as follows.

- The temperature, concentration and velocity fields for isothermal boundary conditions are higher than ramped boundary conditions.
- Isothermal wall temperature and velocity increase by increasing α, while decreases occur in the case of ramped wall temperature and velocity. Contrastingly, concentration increases in both cases.
- Velocities decrease by increasing *R*, *Q* and increase by increasing *Sr*, *Nr*, *φ*.
- Temperatures decrease by increasing *Q* and increase by increasing *Nr*, *φ*.
- Concentrations decrease by increasing *R*, *Nr*, *φ* and increase by increasing *Sr*, *Q*.
- Ramped wall conditions help to manage deviations of temperature, concentration and velocity fields.
- Velocity profile of water–MWCNT is greater than the velocities of water–SWCNT, glycerin–SWCNT and glycerin–WMCNT.
- Temperature of water–SWCNT is higher than the temperatures of water–MWCNT, glycerin–SWCNT and glycerin–WMCNT.
- Concentration profile of glycerin–WMCNT is greater than the concentrations of water–SWCNT, water–MWCNT and glycerin–SWCNT.
- Water–MWCNT has the maximum velocity, while glycerin–SWCNT has the lowest velocity. Because of its increased thermal conductivity, the temperature profiles of water–SWCNT are higher than those of other fluids. Glycerin–MWCNT has a greater concentration than the other fluids under study.
- Skin frictions of water–SWCNT, water–MWCNT, glycerin–SWCNT and glycerin–WMCNT increases by increasing *φ* for both isothermal and ramped conditions on both walls of the channel.
- Nusselt numbers of water–SWCNT, water–MWCNT, glycerin–SWCNT and glycerin–WMCNT increases by increasing *φ* for both isothermal and ramped conditions on both walls of the channel.
- Sherwood numbers of water–SWCNT, water–MWCNT, glycerin–SWCNT and glycerin–WMCNT increase at *y =* 0 and decrease at *y =* 1 by increasing *φ* for both isothermal and ramped conditions on both walls of the channel.

## Figures and Tables

**Table 1:** Thermo-physical characteristics of CNTs and fluids [48].

Material	$\beta \;\times \;10^{-5}$	k	ρ	$c_{p}$	Pr
Glycerin	48	0.286	1259.9	2427	6.78
Water (H_2_O)	21	0.613	997	4179	6.2
SWCNTs	27	6600	2600	427	-
MWCNTs	44	3000	1600	796	-

**Table 2:** Deviation of skin frictions for various values of *φ*.

*φ*	*t*	Water				Glycerin			
		*y* = 0		*y* = 1		*y* = 0		*y* = 1	
		SWCNT	MWCNT	SWCNT	MWCNT	SWCNT	MWCNT	SWCNT	MWCNT
0.01	0.6	0.132	0.268	0.26	0.744	0.284	0.282	0.737	0.738
0.02	0.6	0.133	0.267	0.268	0.768	0.297	0.293	0.754	0.756
0.03	0.6	0.135	0.267	0.277	0.792	0.312	0.306	0.772	0.775
0.04	0.6	0.138	0.268	0.286	0.816	0.327	0.319	0.79	0.795
0.01	1.2	0.081	0.074	0.507	1.447	0.112	0.111	1.433	1.434
0.02	1.2	0.081	0.076	0.523	1.489	0.131	0.129	1.465	1.465
0.03	1.2	0.083	0.08	0.539	1.532	0.151	0.148	1.496	1.498
0.04	1.2	0.086	0.084	0.555	1.576	0.174	0.17	1.529	1.531

**Table 3:** Deviation of Nusselt numbers for various values of *φ*.

*φ*	*t*	Water				Glycerin			
		*y* = 0		*y* = 1		*y* = 0		*y* = 1	
		SWCNT	MWCNT	SWCNT	MWCNT	SWCNT	MWCNT	SWCNT	MWCNT
0.01	0.6	2.716	2.697	0.107	0.103	2.735	2.716	0.11	0.107
0.02	0.6	2.917	2.882	0.15	0.142	2.953	2.918	0.158	0.15
0.03	0.6	3.109	3.058	0.201	0.187	3.161	3.111	0.214	0.2
0.04	0.6	3.294	3.229	0.258	0.237	3.36	3.296	0.278	0.256
0.01	1.2	3.786	3.759	0.293	0.285	3.812	3.786	0.301	0.939
0.02	1.2	4.075	4.023	0.393	0.374	4.125	4.074	0.411	0.392
0.03	1.2	4.352	4.278	0.505	0.474	4.225	4.351	0.534	0.503
0.04	1.2	4.62	4.525	0.627	0.583	4.713	4.62	0.67	0.624

**Table 4:** Deviation of Sherwood numbers for various values of *φ*.

*φ*	*t*	Water				Glycerin			
		*y* = 0		*y* = 1		*y* = 0		*y* = 1	
		SWCNT	MWCNT	SWCNT	MWCNT	SWCNT	MWCNT	SWCNT	MWCNT
0.01	0.6	0.131	0.126	0.69	0.69	0.136	0.095	0.689	0.694
0.02	0.6	0.185	0.176	0.676	0.677	0.192	0.152	0.675	0.68
0.03	0.6	0.228	0.218	0.662	0.664	0.238	0.198	0.661	0.667
0.04	0.6	0.265	0.253	0.648	0.651	0.275	0.237	0.647	0.654
0.01	1.2	0.352	0.344	1.176	1.177	0.308	0.302	1.183	1.185
0.02	1.2	0.422	0.411	1.15	1.152	0.387	0.377	1.157	1.159
0.03	1.2	0.479	0.465	1.125	1.128	0.45	0.439	1.131	1.134
0.04	1.2	0.529	0.511	1.1	1.105	0.502	0.489	1.106	1.111

## Data Availability

Not applicable.

## Nomenclature

$\tilde{C}(\tilde{y_{1}},\tilde{t_{1}})$	Concentration (kgm^−3^)
$\tilde{w}(\tilde{y_{1}},\tilde{t_{1}})$	Velocity (m s^−1^)
$\tilde{T}(\tilde{y_{1}},\tilde{t_{1}})$	Temperature (K)
*D*	Mass diffusivity (m^2^ s^−1^)
*k*	Thermal conductivity (W m^−1^ K^−1^)
g	Gravitational acceleration(m s^−2^)
*c_p_*	Specific heat (J kg^−1^ K^−1^)
$q_{r}$	Radiative heat flux
$Q_{0}$	Heat absorption (W m^−3^K^−1^)
$T_{m}$	Mean fluid temperature
$k_{T}$	Thermal diffusion ratio
$R_{0}$	Chemical reaction (s^−1^)
$k_{1}$	Rosseland absorption coefficient
*G_m_*	Mass Grashof number
$\sigma_{1}$	Stefan-Boltzmann constant
*Pr*	Prandtl number
*Sc*	Schmidt number
*G_r_*	Thermal Grashof number
*Sr*	Soret effect
*Nr*	Thermal radiation
*Nu*	Nusselt number
*R*	Chemical reaction
*Sr*	Soret effect
*Q*	Heat absorption
*S_k_*	Skin friction
*Sh*	Sherwood number
**Greek Symbols**	
*φ*	Nanoparticles volume fraction
*ν*	Kinematic viscosity (m^2^ s^−1^)
*α*	Fractional parameter
*β_T_*	Thermal expansion (K^−1^)
*θ*	Dimensionless temperature
*β_C_*	Mass volumetric (m^3^kg^−1^)
*ρ*	Density (kg m^−3^)
*µ*	Dynamic Viscosity (kg m^−1^ s^−1^)
**Subscript**	
*f*	Fluid
CNTs	Carbon nanotubes
*ssnf*	Nanofluid
